# National Politics’ Role in Developing Primary Health Care Policy for Maternal Health in Papua New Guinea: A Qualitative Document Analysis

**DOI:** 10.9745/GHSP-D-22-00005

**Published:** 2024-10-29

**Authors:** Regina Poima Seki, Judith Daire, Delia Hendrie

**Affiliations:** a School of Population Health, Curtin University, Perth, Western Australia.; b Western Highlands Provincial Health Authority, Mount Hagen, Papua New Guinea.

## Abstract

Politics is one of the critical factors that influence health policy agendas. However, scholarly efforts, especially in low- and middle-income countries, rarely focus on how politics influence health policy agenda-setting. We conducted a qualitative document review to examine the factors that led to developing the free primary health care policy for maternal health in Papua New Guinea. We also discuss mechanisms through which national politics, as an overriding factor, influenced the development of the policy. The review draws on Kingdon’s multiple-stream model for agenda-setting and incorporates theoretical insights from Fox and Reich’s framework for analyzing the politics of health reform for universal health coverage in low- and middle-income countries.

## Abstract (precis)

This article examines the factors and mechanisms that influenced the development of the free primary health care policy for maternal health in Papua New Guinea.

## Teaser

**Key Findings:**

- Although a mix of 3 policy streams—problem stream (persistent poor maternal health outcomes), policy stream (the adopted international policies), and (national) politics stream—influenced the agenda to develop the free primary health care (PHC) policy for maternal health in Papua New Guinea, national politics was observed to have had an overriding role.
- Politics influenced the agenda through 3 main mechanisms: the existing legal frameworks that guide the organization and governance of the health system, which accorded the National Department of Health power and overall responsibility for decision-making; existing national and international policy norms and ideas for addressing maternal health problems; and political interests of key stakeholders to maintain popularity among the electorate.

**Key Implications:**

- Reducing the maternal mortality rate in Papua New Guinea requires integrating the government’s priorities into a fully functional health system.
- A comprehensive health system integration of the government’s priority initiatives to improve maternal health outcomes with the country’s 2020–2030 vision for attaining a 75% reduction in the maternal mortality rate should be adopted to address barriers to access.

## INTRODUCTION

Several theories offer great insights into understanding the central role that politics has in shaping health policies at the global, regional, national, and local levels.^1^^–^^4^ In agenda-setting and policy formulation, politics determine how policymakers recognize and define certain problems and prioritize certain policy solutions over others.^5^ Policymaking, as noted by Reich,^5^ “is not simply a technocratic process based on rational analysis;” instead, it is a profoundly political process. Fox and Reich^6^ argued that politics plays a role at all stages of the policy process through the actors involved, their interests, institutions, and ideologies. Several studies have demonstrated the critical role of politics in influencing policy.^2^^,^^3^^,^^5^^,^^6^ These studies have focused on politics as one of the factors that influence agenda-setting and have considered how certain health priorities receive political attention over others. However, there has been less attention on the mechanisms through which politics influence the policy agenda in health, especially in low- and middle-income countries (LMICs). We examine factors that led to the development of the free primary health care (PHC) policy for maternal health in Papua New Guinea (PNG) and discuss the mechanisms through which national politics influenced the policy development process as an overriding factor.

## METHODS

This document review is part of a case study that evaluated the impact of the free PHC policy on maternal health outcomes in PNG. For this article, we focus on methodology and findings from the document analysis. In the first stage of data collection for the case study, we conducted a document analysis to examine factors that led to the emergence of the free PHC policy for maternal health in PNG. We used a systematic approach for document analysis in health policy research called the READ approach,^7^ guided by a protocol adapted from Altheide^8^ that was customized to fit the study objectives.

In the document review, we identified and examined 15 documents, including legislative documents and health plans, dating from 1957 to 2015, that provided relevant information on agenda-setting and formulation of the free PHC policy for maternal health in PNG and explored the role of politics in the health policymaking process (Table 1).^9^^–^^23^ The relevant documents were accessed from the National Department of Health (NDOH) of PNG, PNG government websites, and institutional websites for international organizations, especially the World Health Organization. Additional documents that provided useful insight into the agenda-setting process in PNG were sought as necessary.

**TABLE 1.** Official Documents Reviewed and Their Relevance to Agenda-Setting for Maternal Health in Papua New Guinea

**Document Name**	**Description and Relevance to Study Objectives**
Constitution of the Independent State of Papua New Guinea^9^	The constitution, amended in 1975 following political independence, provides the national laws, goals, and directive principles and states that all citizens have a right to basic health care.
Public Hospitals (Charges) Act 1972^10^	This Act provides the charges for services at public health facilities in accordance with its regulations.
Public Hospitals (Charges) Regulation 1978^11^	This Act describes the fees charged for services provided at public hospitals and clinics.
Consolidation of the Organic Law on Provincial Governments and Local-Level Governments^12^	The law provides for the provincial administrative arrangements, functions, and responsibilities related to health services provision and the operation of health facilities.
National Health Administration Act 1997^13^	This Act details the administrative arrangements, functions, and responsibilities in relation to provincial health matters between the National Department of Health and the offices of Provincial Administrator and District Administrator for the purposes of Section 80(3) of the Organic Law.
Promoting the Millennium Development Goals in Asia and the Pacific: Meeting the Challenges of Poverty Reduction^14^	The report assesses progress toward meeting the MDGs in the Asia Pacific Region, with measurement of performance against targets, one of which is reducing maternal mortality by three-quarters by 2015.
Papua New Guinea Demographic and Health Survey: 2006 National Report^15^	The report highlighted maternal mortality was high at 733 deaths per 100,000 live births and other MH indicators were poor.
Millennium Development Goals: Second National Progress Summary Report 2009 for Papua New Guinea^16^	The progress report for 2004–2009 indicated that the maternal mortality rate remains at a very high level and collaborative approaches needed to reduce it.
Ministerial Taskforce on Maternal Health in Papua New Guinea Report^17^	The Ministerial taskforce report on MH status highlights issues and barriers to accessing MH services. Maternal mortality remains a major health problem, and maternal indicators of antenatal coverage and supervised deliveries at health facilities are low. MH is stated to be a priority area for the government and the National Department of Health.
Papua New Guinea - Millennium Development Goals Second National Progress Comprehensive Report for Papua New Guinea 2010^18^	This report reviews progress in meeting the national MDG targets during the 1990–2009 period, highlighting maternal mortality and other MH indicators as targets to address to improve MH outcomes.
Papua New Guinea Medium Term Development Plan 2011–2015 “Building the Foundations for Prosperity”^19^	The document detailed a national development action plan, framework, and strategies for 2011–2015, calling for improving and enhancing service delivery to improve quality of life, strengthening PHC to improve MH, and reducing deaths and disease burdens affecting women and children.
National Health Plan 2011–2020: Volume 1 Policies and Strategies^20^	The plan, which provides policy direction to all stakeholders including the public and private sectors, outlines health sector goals, including prioritizing MH and detailing how MH services will be provided and funded.
National Health Plan 2011–2020: Volume 2 Reference Data and National Health Profile^21^	The document outlines the reference data for 22 provinces and 2001–2009 performance data, including MH data.
Health Service Delivery Profile Papua New Guinea 2012^22^	The document discusses the country’s service delivery model and provider network, including an overview of health system components. Maternal mortality is a serious problem with the maternal mortality rate of 733 deaths per 100,000 live births and only 53% of deliveries by health care workers. These issues need to be addressed by the government; strengthening of PHC services is also important.
The Platform for Action^23^	The document details the government’s goals and priorities for 2012–2017, including improving health infrastructure and strengthening PHC including MH, as well as the government’s objective to pursue the vision 2050 directive principles and its commitment to accessible quality health care.

Abbreviations: MDG, Millennium Development Goals; MH, maternal health; PHC, primary health care.

The document review was conducted independently by 3 research team members to achieve consensus. Each document was summarized in a data collection sheet based on its relevance to the study (Table 1).

Data extracted from the documents were organized into categories of information and themes (Table 2) using Kingdon’s agenda-setting model, which posits that policymakers become aware of issues when political developments, problems, and solutions come together to create windows of opportunity.^24^ This framework helps explain how 3 elements or “streams”—problems, politics, and policies— affect the dynamics of policy agenda-setting. This model challenges assumptions that health issues rise onto policy agendas solely through rational deliberation and careful consideration of evidence.^3^ Although the framework identifies politics as an important aspect of the policy process, it does not offer insights into the mechanisms through which politics influences the policy agenda. Thus, we used theoretical insights from Fox and Reich^6^ to discuss the mechanisms through which national politics influenced the agenda of the free PHC policy for maternal health in PNG. Fox and Reich^6^ posited that politics influence policy from agenda-setting to its outcomes through the institutions (both formal and informal) that affect how policies are made, the interests of stakeholders involved, ideas about existing policy solutions, and predominant ideologies reflecting prevailing policy concepts and paradigms.

**TABLE 2.** Identified Themes and Categories of Information Extracted From Reviewed Documents

**Themes and Categories of Information**	**Extracted Information and Source**
**Policy context**	
Social values underpinning government policies	Strong social norms and value for women’s health underpin government policies, laws, and regulations.^18^
Changes in politics	Colonial government from 1906 to self-government in 1973, then to independence in 1975.^10^ PNG gained political independence and democracy in 1975.^10^ Change of government in 2012, new prime minister.^24^
Health sector reforms	Decentralization of government roles and responsibilities affected funding for PHC facilities.^18^ Funding for health and PHC services decreased.^18^
Health system challenges and provider-entrenched practices	Inadequate funding and poor reinforcement of fee exemption policy for maternal health services.^18^^,^^23^
Competition for funding priorities	More political attention for infectious diseases compared to maternal and reproductive health.
**Poor maternal health outcomes (Problem stream)**	
High maternal mortality	Demographic and Health Survey data estimates the maternity mortality ratio to be 370 deaths per 100,000 live births in 1996 and 733 in 2006; modeled estimate, calculated by international bodies, suggests 230 for 2010.^16^^,^^17^^,^^19^
Utilization of maternal health	Skilled birth attendance in 2005 was 41% coverage, in 2008 and 2010 was 51%.^17^^–^^19^^,^^21^^,^^22^ Antenatal care visits had 58% coverage in 2000, 59% in 2005, 80% in 2010.^17^^–^^19^^,^^21^^,^^22^ Postnatal care visits limited or no data on postnatal visits.^18^^,^^21^^,^^22^
**Global events and/or policy norms (Policy stream)**	
International policy norms or priorities, obligations, or agreements	Before PHC concept, maternal and child health services were provided as vertical programs. After adopting PHC concept, maternal and child health services became essential at all PHC facilities.^15^^–^^22^ PHC concept was introduced in 1978 by World Health Organization and all countries were urged to adopt and formulate national policies and strategies and plans to deliver adequate low cost health services to all.^15^^–^^22^ PNG also adopted the international Safe Motherhood Initiative; it was implemented as a project spearheaded by the United Nations Population Fund.^15^^–^^22^ As a signatory to the International Conference on Population and Development in 1994, PNG broadened its maternal health to include women’s rights to sexual and reproductive health.^15^^–^^22^ PNG was a signatory to the declaration of Millennium Development Goals for 2000–2015, Universal Health Coverage in 2010 and Sustainable Development Goals in 2015.^15^^–^^22^
**National politics (Politics stream)**	
Free maternal health services	Free PHC services was on government agenda since colonial period.^21^^,^^22^ Universal health care concept from international polices incorporated into government policies.^21^^,^^22^ Recommendations from maternal health review.^18^ A political agenda for the current government and free services provided for in government policies.^18^
User fee	Only applicable in tertiary health facilities both during colonial and post-colonial periods.^18^^,^^21^^,^^22^
Fee exemption for maternal health	Provided for in national health plan of 2001–2010 but health providers still charged user fees.^21^^,^^22^

Abbreviations: PHC, primary health care; PNG, Papua New Guinea.

## RESULTS

We discuss key themes from the document review, starting with the context within which the free PHC policy for maternal health was developed, followed by an examination of factors that led to the development of the PHC policy for maternal health in PNG.

### Policy Context in Papua New Guinea

The fundamental premise of the free PHC policy in PNG was to offset user fees routinely collected at health facilities by providing subsidy payments from the NDOH^25^ and to promote free access to PHC services at service delivery points. The provision of free maternal health care services has been on the government’s agenda since the colonial government. As early as 1957, the PNG government’s health legislation was founded on the principles of the right for all citizens to receive basic health care services and the value placed on the well-being of women and children.^9^ Such principles underpinned the government’s drive to provide access to PHC services and fee exemption policies for maternal health services. It was never legal for PHC facilities to charge user fees. The hospital user charge policy of 1972, which was changed in 1978 to a public hospital charges regulation, allowed hospitals to charge user fees for tertiary care only.^10^^,^^11^^,^^26^

The fundamental premise of the free PHC policy in PNG was to offset user fees routinely collected at health facilities by providing subsidy payments from the NDOH.

After gaining independence in 1975, the PNG government implemented both structural and regulatory reforms between the late 1970s and early 1990s as part of the changes needed to reflect political administrative independence. In 1995, the government introduced the Consolidation of the Organic Law on Provincial Governments and Local-Level Governments to decentralize the management and administration of government services,^12^ followed by the National Health Administration Act of 1997 (NHAA).^13^ These resulted in the separation of administrative authorities for rural health services and hospitals, which were previously vertical entities. Rural health services were decentralized to the local government, whereas hospital planning, management, and administration were delegated to provinces.^27^ However, these reforms presented significant challenges because the functional roles and responsibilities of provinces and local governments were poorly defined, leading to poor resource allocation, inadequate funding, lack of coordination, and inefficient management of health care services, including maternal health.^28^

To supplement allocated budgets from the NDOH, PHC facilities started charging user fees. Because there was no national legislation on the establishment of user fees for PHC services, the practice of collecting user fees in PHC facilities was unregulated.^28^ The practice became common across most facilities after further decentralization of government roles and responsibilities to districts and local governments, as stipulated in the NHAA.^13^^,^^29^

Inadequate funding for maternal health and reproductive health became more profound due to competing priorities. Maternal health was noted to have received less political attention in the 1990s due to the disease control programs funded by global health initiatives.^28^ Although maternal health care services became fee exempted under the PNG National Health Plan 2011–2020,^20^ most PHC facilities continued to charge fees because of the persistent inadequate funding from the NDOH. Insufficient funding for maternal health at the PHC level was associated with poor health outcomes, as reported in several documents.^15^^–^^17^ For example, the Maternal Health Task Force report of 2009 noted that the escalating poor maternal health indicators could be associated with poor access to services due to user fee charges. In response to poor health outcomes, such as high maternal mortality, the NDOH developed the National Health Plan 2010–2020 to strengthen the PHC delivery system, especially in rural areas where the majority of the population lives.^20^ Additionally, the NDOH made improving maternal health 1 of its 8 key health priorities.

In 2012, the newly elected Prime Minister placed free PHC services on the government’s political agenda, expressed through the Alotau Accord of 2012, which became the foundation for developing the free PHC policy to provide free maternal health care services.^23^ The Figure outlines the policy events in PNG leading up to the development of the free PHC policy for maternal health.

**FIGURE:**
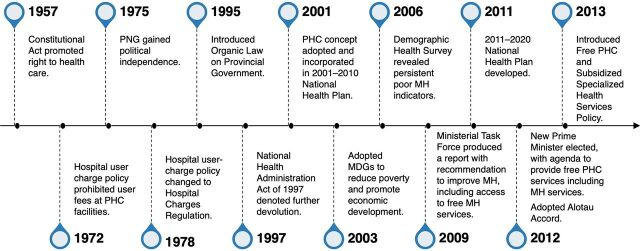
Timeline Leading to the Introduction of the Free PHC Policy in Papua New Guinea in 2013 Abbreviations: MDG, Millennium Development Goals; MH, maternal health; PHC, primary health care; PNG, Papua New Guinea.

### Factors Leading to the Free Primary Health Care Policy Development

The development of free PHC policy for maternal health was influenced by all 3 policy streams in Kingdon’s agenda-setting model: the problem stream (persistent poor maternal health outcomes), the policy stream (the adopted international policies), and the (national) politics stream.

#### Persistent Poor Maternal Health Outcomes

In line with human rights and social values of advancing women’s health, as reflected in several government documents, persistent poor maternal health outcomes have been the driving force for the political attention on maternal health in PNG for the last 3 decades. Most documents reviewed identified maternal mortality as a health priority requiring the government’s attention. This was particularly emphasized in the Maternal Health Task Force Report,^17^ National Department of Health Sector Annual Performance Report 2008–2012,^30^ and the Millennium Development Goal progress reports of 2009^16^ and 2010,^18^ which highlighted that limited progress had been made toward improving maternal health. The high maternal mortality rate was viewed as an important indicator of gender inequality and the poor performance of the national health system as a whole.^16^^,^^18^

The goal of reducing maternal death was also noted in all health sector plans since 2010 as the government’s aspiration of “saving mothers.”^23^ The persistent high maternal mortality rate reflected not only political failure to steward publicly held health values about women’s health and well-being but also failure to comply with internationally agreed policy norms on improving maternal health outcomes to which the government was a member.^31^

#### Influence of International Policy Norms

As a state party to United Nations agencies, the government’s development of maternal health policies in line with the international policy norms to address maternal mortality can be traced to the introduction of the PHC concept at the Alma Ata conference in 1979, calling for universal access to basic health care services for all population groups.^32^ In line with this concept, PNG developed policies that streamlined basic health care services for reproductive and childbearing population groups to operationalize the Safe Motherhood Initiative of 1987 and the International Conference on Population and Development in 1994, which both unanimously advocated for universal access to health care as a right for childbearing women. Although the PNG government adopted these international policy norms, PNG health facilities continued charging user fees, hindering the government’s pursuit of universal health coverage (UHC) through PHC.

This practice of charging user fees was strengthened by the World Bank Bamako Initiative introduced in 1993 that supported charging user fees as a means of cost recovery.^33^ As of 1997, user fees were charged by health facilities for operational costs and often levied by the government to supplement health budgets.^34^

The impact of user fees as a barrier for poor and vulnerable populations to access basic health care services is well known worldwide.^35^ The revitalization of the PHC concept and UHC, as shaped by global economic and health policy goals, sustained the agenda of making quality health care services accessible to everyone regardless of the ability to pay. Additionally, the adoption of the Millennium Development Goals played a critical role in sustaining maternal health as a priority on the policy agenda in both national and international contexts.^3^ To that end, the National Health Plan 2011–2020 aimed to strengthen PHC for all and improve service delivery for the rural majority and urban disadvantaged.^20^ As in many low- and middle-income countries, PNG adopted many of the international policy norms to improve maternal health, which led to the development of policies on free PHC, including maternal health care services. However, national politics seem to have played an overriding role in influencing the development of the free PHC policy for maternal health.

#### National Politics

National politics played a key role in influencing the diffusion and operationalization of international policies related to maternal health and universal health access. Access to health care services, especially among women and low socioeconomic groups, had been on the political agenda since PNG gained independence in 1975.^36^ For example, as observed by PNG’s Independent Health System Review, successive governments strived to ensure accessible and affordable services to the entire population and particularly women, children, and at-risk population groups.^36^ However, the free PHC policy only came to fruition in 2013, during the first O’Neill-Dion term in government.

Considered a major social policy reform, providing free PHC across PNG was a pre-election campaign agenda item for the O’Neill-Dion party. After taking office in 2012, the new O’Neill-Dion government announced in the Alotau Accord^23^ that it would fulfill its political commitment to accessible and affordable health care by providing free PHC and subsidized specialist services.^26^ As the overall responsible sector, the NDOH showed full support by acknowledging that user charges prevented the poorest people of PNG from enjoying the right to access PHC. To that end, the Prime Minister and other senior politicians publicly declared their commitment to providing free PHC services to reduce maternal mortality.^26^

Before the O’Neill-Dion government, several reports had highlighted maternal mortality as a problem and proposed solutions.^16^^,^^17^^,^^30^ However, the Health Sector Performance Annual Review 2008–2012, which showed a snapshot of 29 health indicators of provinces, showed very minimal change to the overall policy outcomes.^37^ The motivation behind the O’Neill-Dion government to change the policy trajectory for maternal health by providing free PHC drew upon the government’s tuition fee-free policy for all levels of schooling from 2012. The political aspiration to provide free health services was well captured in the 2012 Alotau Accord and was popular among the ruling party politicians, as they saw it resonated well with the electorate.^23^

### Mechanisms Through Which National Politics Influenced Policy Development

The mechanisms through which national politics influenced the free PHC policy for maternal health are consistent with the processes discussed by Fox and Reich^6^ in their framework to analyze health reforms aimed at achieving UHC. These included legal frameworks underpinning the overall organization and governance of the health system in PNG that accorded the NDOH power and overall responsibility for decision-making, existing national and international policy ideas about solutions for addressing maternal health problems, and political interests of key stakeholders to maintain popularity among the electorate.

#### Legal Framework Underpinning Health System Governance

In PNG, the NHAA^13^ provides the legal framework for linking and consolidating the functions of all levels of government and other agencies involved in health care delivery.^13^^,^^38^ Within this legal framework, the NDOH has an overarching governance role in the health care system to oversee the establishment, maintenance, and development of the health care system by setting policies and standards, providing technical support for the operation of health facilities, and being responsible for health service delivery.^36^^,^^38^ The NDOH also oversees the management of public hospitals in accordance with the Public Hospitals Act of 1972 and the rollout of legislative changes in the Provincial Health Authority Act.^39^ The legal framework allows the Prime Minister to appoint ministerial positions, including for the NDOH. Hence, the political nature of the Minister of Health’s position accorded the new government a platform to transform free PHC policy from political to a more formal government policy agenda for priority action.

The NHAA also determined key stakeholders involved in developing the free PHC policy. Along with the Minister of Health, the Minister of Finance, Secretary for Health, and Deputy Secretary of Health, and other senior public servants, who were part of the government bureaucracy as key decision-makers, had a significant role because of their positions. These positions were also politically appointed and expected to act in line with the ruling party’s agenda. Further, the NHAA determined institutional bodies from which government decision-makers obtained advice on various government policy matters. In the case of the free PHC policy, the NHAA established the National Health Board, which advises the Minister on policy matters relating to health, including the formulation, extension, amendment, and replacement of the National Health Plan and approval of the National Health Standards for purposes of implementing the plan.^38^ Most individuals appointed as advisory board members, such as health advisory boards at national, provincial, local government, and district levels, follow political loyalties and patronage rather than technical expertise to execute high-level policy responsibilities. As such, it was easy for the government decision-makers to mobilize political support to develop the free PHC policy.^36^

#### Policy Ideas About Addressing Poor Maternal Health Outcomes

Policy ideas about solutions to address persistently poor maternal health outcomes, both from the national and international community, were noted in the National Health Plan of 2011 and the Ministerial Task Force report.^17^ Both documents identified universal coverage of maternal health care services through free PHC as one of the key priorities to improve maternal health outcomes in PNG.^17^^,^^20^

The O’Neil-Dion government operationalized free PHC through its political agenda as stipulated in the Alotau Accord,^23^ which incorporated the international policy norms for improving maternal health and achieving UHC and health-related goals from the international community. The 2009 Ministerial Task Force on Maternal Health^17^ is reported to have informed the Alotau Accord through which the government planned to adopt a platform to improve access to health services. Promoted as a formal statement of government policy, access to free health care services was identified as one of the government’s priorities. As the government embarked on this new platform for action, it pledged to maintain its commitment to accessible and affordable health care by providing free PHC and subsidized specialist services.^23^ As a political agenda for the ruling party, the Alotau Accord was the basis for developing free PHC service packages, including maternal health care. The free PHC policy is described as a tool to implement the Alotau Accord commitment to health with a system-wide approach across facility levels in 2 stages.^25^ Hence, the political system became the medium through which the existing policy ideas of UHC through free PHC and other strategies related to achieving the Millennium Development Goals and the PHC concept gained policy agenda status and was operationalized.

## DISCUSSION

We provide insights into the role of politics as an influence on maternal health, gaining momentum in the political agenda. Based on our analysis, we see a convergence of efforts that revealed that a mix of all 3 policy streams in Kingdon’s agenda-setting model influenced the development of the free PHC policy for maternal health.

Poor maternal health outcomes that persist in low- and middle-income countries with limited resources fight for political attention, particularly when it comes to agenda-setting. The emphasis on reducing the maternal mortality ratio is driven by many factors, including Millennium Development Goals, high maternal mortality, inequality in maternal health service utilization, and outcomes. It is clear that the maternal health issue profoundly influenced the agenda to gain political priority.

Within the context of a wider evaluation, international actors and policies influence countries to first pursue the cause of maternal mortality reduction. Policy actors were from both government and nongovernmental organizations and agencies and were all committed to common causes.^4^ The international consensus to reduce maternal mortality laid the foundation for its inclusion among the health and development priorities that emerged as both a global and national priority.

In this analysis, we understand that policy is both a deliberate and a purposive act and subject to contestation. Although health policy can be understood as decisions, plans, and actions that are undertaken to achieve specific health care goals within a society, it is also, in essence, about process and power. As stated by Walt and Gilson,^40^ health policy is concerned with who influences whom in the making of policy and how it happens.

However, national politics seem to have played an overriding role because the free PHC policy was developed at a prime time for the newly elected government to demonstrate its commitment to improving access to health care through free PHC services. Access to health care services, especially among women and low socioeconomic groups, has been on the political agenda since PNG gained independence in 1975. Successive governments strived to ensure accessible and affordable services to the entire population. However, the free PHC policy only came to fruition in 2013 during the first O’Neill-Dion political term in government. The mechanisms through which national politics influenced the free PHC policy for maternal health in PNG included the legal acts underpinning the overall organization and governance of the health system, which promoted the political appointment of senior government decision-makers and members of health advisory boards across all health system governance levels. The political nature of the ministerial and other senior decision-making positions within the NDOH gave the new ruling party the authority to prioritize the free PHC policy agenda and mobilize the needed political support to develop the free PHC policy.

The mechanisms through which national politics influenced the free PHC policy for maternal health in PNG included the legal acts underpinning the overall organization and governance of the health system.

Second, existing institutional legal frameworks also determined the key stakeholders involved in decision-making and policy development who predominantly had political interests. Stakeholder engagement fosters effective policy development. PNG is also privileged to have a wide range of stakeholders supporting the government in implementing development initiatives in the country, including maternal mortality reduction. The political interests of stakeholders to advance free PHC policy were further reinforced through their appointment of advisory board members based on political loyalties and patronage instead of technical expertise to execute the high-level policy responsibilities. As such, it became easy for the government decision-makers to mobilize political support for policy actions like the free PHC policy.

Third, policy ideas like free PHC to address persistent poor maternal health outcomes already existed both at national and international levels, which the O’Neil government adopted as the party’s political agenda.^23^

### Limitations

Insights from this study are limited to 1 policy area, namely agenda-setting. For more generalized insights, an analysis of the political influence on several policies and mechanisms through which such policies were politically influenced is needed. In addition, future research could also focus on how politics at the agenda-setting stage affects policy design, tools, implementation, and outcomes.

The scope of analysis did not include other enabling factors like the role of non-state actors within the country, given that nongovernmental organizations are the dominant health service providers in rural areas where most poor maternal health outcomes are associated with complications in maternal health care outside urban areas.

Future research can investigate how reforms around free PHC policy influenced nongovernmental health provider practices. Studies have shown that health providers still charge user fees for essential PHC services even after the free PHC policy was developed. Finally, future research can investigate how the free PHC policy in PNG is linked to UHC in the context of decentralized policy established by the Organic Laws and the extent to which establishment of essential PHC packages of services has improved health coverage, particularly in provinces with the largest burden of disease.

## Notes

**Peer Reviewed**

**First Published Online:** September 24, 2024.
